# Man-in-the-Barrel Syndrome Secondary to Idiopathic Acute Anterior Spinal Artery Infarction

**DOI:** 10.7759/cureus.41549

**Published:** 2023-07-08

**Authors:** Sarah Baroud, Fatima Al Zaabi, Waqar H Gaba, Mohammed El Lahawi

**Affiliations:** 1 Internal Medicine, Sheikh Khalifa Medical City, Abu Dhabi, ARE; 2 Neurology, Sheikh Khalifa Medical City, Abu Dhabi, ARE

**Keywords:** acute anterior spinal artery infarction, owl's eyes sign, cervical spine disorders, brachial diplegia, man-in-the-barrel syndrome

## Abstract

A 52-year-old female presented to our hospital with an acute history of isolated bilateral arm weakness. An MRI of the cervical spine confirmed an acute anterior spinal artery infarction. Further investigations to determine a specific etiology were unremarkable, leading to a diagnosis of idiopathic anterior spinal artery infarction consistent with Man-in-the-Barrel syndrome.

## Introduction

Man-in-the-barrel syndrome (MIBS) is a neurological condition characterized by bilateral arm (brachial) paresis with preserved lower extremity (crural) and bulbar (medullary) strength. The term is denoted by the patient's diminished ability to move their arms in response to a stimulus and carry out functional activities related to raising their arms, drawing a parallel to the feeling of being 'stuck in a barrel'. Several other descriptive terms exist including ‘person-in-the-barrel syndrome’, ‘brachial diplegia’, ‘flail arm syndrome’, ‘distal field infarction’, and ‘cruciate paralysis’.

MIBS is caused by bilateral symmetric injury to the motor fibers specifically responsible for arm movement. It was originally attributed to systemic hypoperfusion, such as in cardiac arrest, resulting in bilateral watershed infarcts between the middle and anterior cerebral artery distributions. However, other pathophysiological mechanisms have been reported including multifocal injury to the cerebral cortex, corona radiata, internal capsule, basal ganglia, brainstem, cervical spinal cord, pyramidal cortico-spinal pathway, brachial plexuses, peripheral nerves, neuromuscular junction, and anterior horn cells [1-3]. In a literature review conducted by Soares et al., it was reported that up to 33% of individuals diagnosed with MIBS were females [4].

In this report, we present a rare case of MIBS that occurred as a result of an idiopathic anterior spinal cord infarction.

## Case presentation

A 52-year-old right-handed Asian female with a history of conservatively managed cervical spondylosis presented to the emergency department with bilateral arm weakness which developed over the last few days and worsened on the day of presentation. She reported an inability to raise her arms, describing it as though she were confined within a tight space. Her symptoms were associated with a longstanding history of bilateral arm paresthesia and posterior cervical neck pain which worsened during this period. There was no facial weakness or visual symptoms.

Other medical history included hypertension controlled on oral medications, a penicillin allergy leading to severe anaphylactic reaction, and a surgical appendectomy. She denied any recent fever, upper respiratory infection symptoms, history of trauma, or head/neck injury. Two months prior to her presentation, she received a rabies vaccine following a stray cat bite with an uncomplicated course; there was no other recent vaccination history. She was a non-smoker and did not drink alcohol or use illicit drugs. She used to work as a cabin crew in an airline but was unemployed since the coronavirus disease 2019 (COVID-19) pandemic. She was single and not sexually active at the time of presentation or on any contraceptive treatment.

On presentation, her vital signs were within normal limits. She had a BMI of 27.3, indicating being slightly overweight. Systemic examination of the cardiovascular, respiratory, abdominal, and integumentary systems revealed no abnormalities. Initial neurological examination showed higher mental function and cranial nerves were intact. Examination of the arms showed reduced tone and global reduced power (3/5). Bilateral shoulder abduction was restricted, and she had difficulty raising her arms. There was no muscle wasting or fasciculation. Reflexes were reduced and sensations of pain, temperature, and light touch were diminished along the C5 dermatome of her right arm. Proprioception, vibration, fine touch, and two-point discrimination were otherwise intact across all other sensory levels. Examination of the legs, including complete sensation, tone, power, and reflexes, was normal and planters were downgoing bilaterally. There were no cerebellar signs. The head was normocephalic and atraumatic. There was no palpable thyroid swelling, enlarged cervical lymph nodes, or neck mass. Bilateral carotid pulses were intact, and peripheral pulses were palpable and confirmed using a handheld Doppler. Examination of the back was unremarkable.

Early into her hospitalization, the follow-up neurological examination was notable for new findings of global reduced power (4/5) in her legs, increased reflexes (without clonus), and equivocal plantar responses. Her gait became unsteady, necessitating assistance while walking, for which she was referred to the inpatient rehabilitation team. Moreover, during her stay, she developed urinary and bowel incontinence necessitating the placement of an indwelling Foley catheter.

Investigations

Her baseline laboratory tests, including a complete blood count, serum creatinine, urea, electrolytes, thyroid function test, creatine kinase level, C-reactive protein, and venous blood gas, were unremarkable. Her baseline ECG showed normal sinus rhythm. Chest X-ray was unremarkable. Computerized tomography (CT) scan of the head and neck without contrast was negative for any acute intracranial abnormality. However, it did indicate the presence of degenerative cervical disc disease at the C5-C6 level without any evidence of spinal cord compression or the existence of bony lesions in the cervical vertebrae.

Additionally, a CT angiography (CTA) head and neck revealed mild narrowing of the right proximal cervical internal carotid artery, possibly due to the presence of a soft plaque, as well as a hypoplastic right vertebral artery, and redemonstration of the degenerative changes at C5-C6. There was no evidence of significant atherosclerosis, aneurysm, irregularity, filling defects, stenosis, or dissection of the major vessels. 

Further imaging was ordered at the onset of her progressing symptoms. Magnetic resonance imaging (MRI) and magnetic resonance angiography (MRA) of the head without contrast were normal and showed no evidence of white matter disease on diffusion imaging, including no acute or subacute infarct, and major vessels were grossly patent.

**Figure 1:**
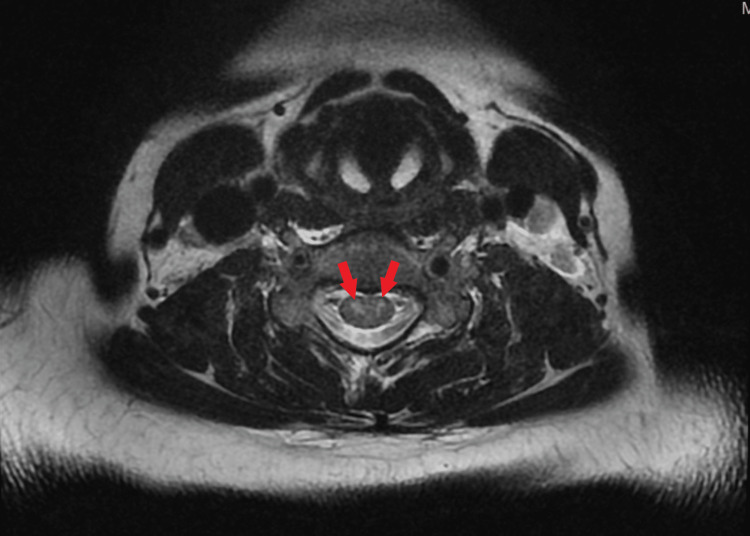
MRI of the cervical spine (axial T2 view) showing bilateral high T2 signal intensity known as 'Owl's eyes'

**Figure 2:**
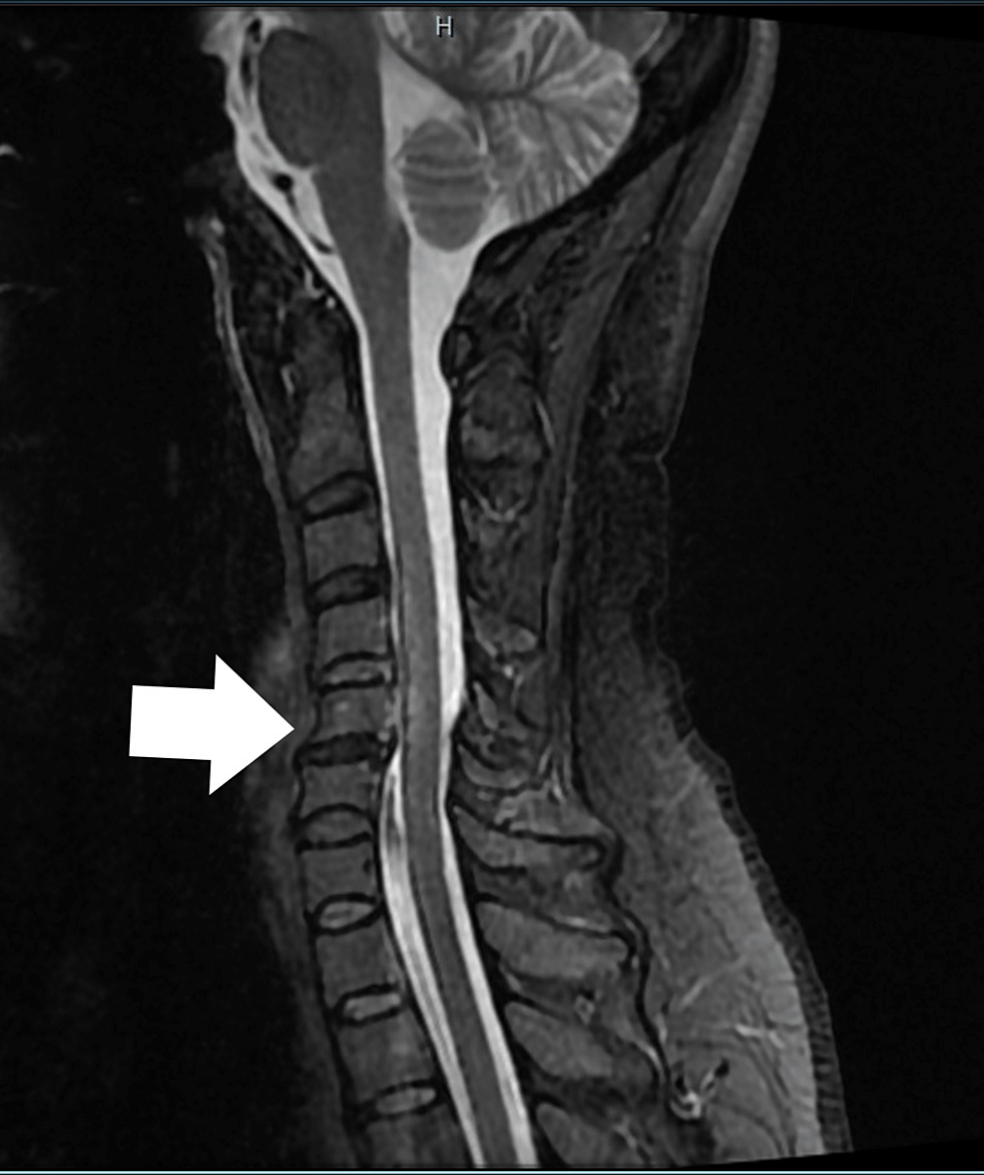
MRI of the cervical spine, sagittal T2 view showing degenerative disc changes in C5-C6 without cord compression

MRI multiplanar T1 and T2 imaging of the cervical and thoracic spine without contrast demonstrated bilateral lateral grey matter spinal cord patchy but confluent T2 signal change clearly in the anterior grey matter with relative sparing of the dorsal cord and dorsal grey matter with 'Owl's eyes' appearance on axial T2 imaging (Figure 1), constellation of findings consistent with long segmental cervical cord acute infarct extending from C2-C3 to C5-C6. Bilateral vertebral arteries were intact with no gross evidence for dissection. Moderate disc degeneration was again observed at the C5-C6 level, accompanied by mild spondylosis (Figure 2). Moreover, a posterior disc bulging with asymmetry to the right was identified, resulting in mild compression of the ventral thecal sac and the medial foraminal/nerve root.

Further investigations (Table 1), including a transthoracic echocardiogram with a bubble study, were unremarkable.

**Table 1:** Laboratory investigations PCR: polymerase chain reaction; AI: antibody index; MPL: IgM phospholipid units; GPL: IgG phospholipid units; IU/U: international unit; ml: milliliter; pmol: picomoles per liter; umol: micromole per liter

Investigation	Value/Finding:	Lab reference
Serum Neuromyelitis Optica (NMO) IgG Antibodies	Negative	Negative
Serum Anti-Myelin Oligodendrocyte Glycoprotein (MOG) Antibodies	Negative	Negative
Lupus Anticoagulant Screen	31.7	0.00 - 44.0 (seconds)
Anti-Double Stranded (DS) DNA Antibodies	<1	<=4 (IU/mL)
Antichromatin Antibodies	<0.2	0.0-0.9 (AI)
Antinuclear-Ribonucleoprotein (RNP) Antibodies	<0.2	0.0-0.9 (AI)
Antiscleroderma-70 (Scl-70) Antibodies	<0.2	0.0-0.9 (AI)
Anti-Smith (Sm) Antibodies	<0.2	0.0-0.9 (AI)
Anti-Ro (SS-A) Antibodies	<0.2	0.0-0.9 (AI)
Anti-La (SS-B) Antibodies	<0.2	0.0-0.9 (AI)
SM-RNP Antibodies	<0.2	0.0-0.9 (AI)
Anti-Jo-1 Antibodies	<0.2	0.0-0.9 (AI)
Anticentromere-B Antibodies	<0.2	0.0-0.9 (AI)
Anti-ribosomal P (anti-Rib-P) Antibodies	<0.2	0.0-0.9 (AI)
Anti-Proteinase 3 Antibodies	<0.2	0.0-0.9 (AI)
Antinuclear Antibodies (ANA)	Negative	Negative
Anti-Myeloperoxidase (MPO) Antibodies	<0.2	0.0-0.9 (AI)
Beta-2 Glycoprotein 1 IgM Antibodies	1.9	0.0-19.9 (U/mL)
Beta-2 Glycoprotein 1 IgG Antibodies	<1.4	0.0-19.9 (U/mL)
Cardiolipin IgM Antibodies	2.1	0.0-19.9 (MPL U/mL)
Cardiolipin IgG Antibodies	<1.6	0.0-19.9 (GPL U/mL)
Vitamin B12 Level	546.3	145.0-569.0 (pmol/L)
Homocysteine Level	6.2	0.0-15.0 (umol/L)
Angiotensin-Converting Enzyme Level	23	14-82 (U/L)
Methyleneetrahydrofolate Reductase (MTHFR) PCR	No Mutation Detected	No Mutation Detected
Factor V Leiden (R506Q) Mutation	No Mutation Detected	No Mutation Detected
Prothrombin Factor II (G20210A) Mutation	No Mutation Detected	Mutation Detected
Protein S Free Antigen, Plasma	95	57-157 (%)
Protein C Activity, Plasma	157	73-180 (%)
Antithrombin Activity, Plasma	119	75-135 (%)

A diagnosis of idiopathic acute anterior spinal artery infarction was made and our patient was initiated on a daily regimen of aspirin 100mg, a high-intensity statin (atorvastatin 40mg), and prophylactic subcutaneous injections of enoxaparin. No further intervention was advised by the Neurology team.

She was discharged from the hospital after a 10-day stay. At the time of discharge, she had demonstrated notable improvement; she could walk with support using a walker and received training for intermittent-self catheterization to address her urinary retention. Her Modified Rankin Score Disability score was determined to be 3, indicating a moderate level of disability. Referrals were made for outpatient physiotherapy and the urology clinic to ensure continued care and facilitate rehabilitation.

## Discussion

MIBS is a rare neurological syndrome often associated with cerebral hypoperfusion, which can occur in conditions such as systemic hypotension, cardiac arrest, following major cardiac surgery, or rapid correction of hypertension, and can lead to bilateral ischemic watershed strokes [5-7]. In addition to the aforementioned causes, supratentorial injuries can also be caused by cerebral metastases [8] and closed head injuries [9]. Infratentorial injuries, although less common, can be attributed to conditions such as pontine ischemia, extrapontine, and central pontine myelinolysis due to hyponatremia, anterior spinal artery ischemia/infarction in cervical spinal cord disorders, amyotrophic lateral sclerosis, bilateral brachial plexus injury, and myasthenia gravis [10-18]. It is worth noting that up to one-third of individuals affected by these conditions have been reported to be females [4].

Approximately 10% of ischemic strokes are attributed to watershed infarctions [1], whereas spinal cord infarction accounts for approximately 1% of cases [15]. The classical presentation of spinal cord infarction includes tetraplegia with symmetric upper extremity weakness, dissociated sensory loss, and bowel and bladder dysfunction. This condition can occur due to inadequate blood flow through the anterior spinal artery, which may be caused by various factors such as vertebral artery thrombosis, vertebral artery dissection, subclavian artery stenosis, or medullary infarction resulting from anterior spinal artery occlusion. The role of chronic degenerative changes in causing acute spinal cord ischemia is still debated [12,14]. Involvement of the anterior spinal artery and frequently the anterior horn cells represent most cases of cervical spinal cord disorders causing MIBS, and the most common cause is idiopathic. The diagnosis of MIBS secondary to anterior spinal artery infarction requires a comprehensive evaluation, which involves taking a thorough medical history, conducting a complete neurological physical examination, and localizing the lesion through cervical spine MRI. The 'owl's eyes sign' seen on MRI, represented by two bright dots indicating high T2 signal intensity on axial imaging, can be seen in anterior spinal artery infarctions. However, other potential differential diagnoses to consider would be demyelinating disease specifically neuromyelitis optica spectrum disorder, viral myelopathy, chronic compressive myelopathy, and less likely toxic or nutritional deficiency myelopathy [19,20]. In our case, the combination of clinical and radiological findings, along with the exclusion of other possible causes through a comprehensive investigation panel, and the valuable input from the Neurology team, all together supported the diagnosis of idiopathic acute anterior spinal artery infarction.

Treatment options for MIBS secondary to anterior spinal artery ischemia may include initiating antithrombotic medications, considering surgical intervention for cases amenable to neurosurgical procedures, and implementing intensive physical and occupational therapy [1]. The prognosis of MIBS varies depending on factors such as the type, degree, and location of the lesion, as well as the promptness of intervention. Favorable prognostic factors include non-comatose status, presence of extracerebral conditions, single sulcal artery occlusion, intact proprioceptive sensation indicating spared dorsal columns, smaller infarcts with the owl's eyes appearance on MRI, and MIBS due to myasthenia gravis, which can be fully reversible with appropriate treatment [1,5,19].

Our patient's presentation is a rare occurrence of MIBS resulting from idiopathic cervical anterior spinal artery infarction, which has been seldom reported in the medical literature. Moreover, her initial presentation with incomplete isolated brachial diplegia is a rare and atypical pattern [14]. However, due to the diligent efforts of the primary medical team, which involved a thorough evaluation of her medical history and physical examination, along with appropriate consultation with Neurology and the prompt utilization of MRI, she was diagnosed early in the course of her disease. This timely treatment provided her with a better chance of a favorable outcome. The comprehensive approach taken in her management highlights the importance of timely and appropriate medical intervention in cases of MIBS.

## Conclusions

MIBS caused by idiopathic anterior spinal cord infarction is a rare neurological condition with a variable prognosis. Accurate diagnosis relies on a comprehensive assessment with a detailed clinical evaluation and timely radiological imaging. Prompt identification and intervention can lead to improved outcomes for patients with MIBS.
